# Predictive value of red blood cell distribution width in critically ill patients with acute respiratory distress syndrome: A meta-analysis

**DOI:** 10.1097/MD.0000000000042701

**Published:** 2025-06-06

**Authors:** Fei-Fei Hou, Yong Song, Wei-Na Du, Bei-Bei Wang, Qiong Wang, Qiong Wu, Li-Na Yan, Xin Chen

**Affiliations:** a Department of Intensive Care Unit, Inner Mongolia Medical University Affiliated Hospital, Hohhot, Inner Mongolia, P.R. China; b Department of Intensive Care Unit, Zibo Traditional Chinese Medicine Hospital, Zibo, Shandong, P.R. China; c Department of Critical Care Medicine, Huantai County People’s Hospital, Zibo, Shandong, P.R. China; d Department of Cardiology, The First People’s Hospital of Jinzhong, Jinzhong, Shanxi, P.R. China; e Department of Plastic Surgery and Burn, Hohhot First Hospital, Hohhot, Inner Mongolia, P.R. China.

**Keywords:** acute respiratory distress syndrome, meta-analysis, predictive value, red blood cell distribution width

## Abstract

**Background:**

Critically ill patients with acute respiratory distress syndrome (ARDS) are one of the leading causes of death worldwide. Although a number of relevant predictors of ARDS have been identified, the current predictors are not satisfactory. Recent studies have revealed the predictive value of red blood cell distribution width (RDW) for ARDS. Therefore, we conducted the first meta-analysis to explore the predictive value of RDW in critically ill patients with ARDS.

**Methods:**

A literature search was conducted to identify relevant observational studies from January 1, 2000, to August 1, 2024. Eligible studies were screened and data were extracted. The standardized mean difference (SMD) with 95% confidence interval (CI) of the RDW levels for each study were combined under the random-effect model.

**Results:**

Ten articles with a total of 2252 participants were included in the study. Elevated RDW levels on admission was significantly associated with significantly associated with an increased risk of ARDS morbidity (SMD = 1.09; 95% CI = 0.35–1.82; *P* = .004), and also significantly associated with an increased risk of ARDS mortality (SMD = 0.73; 95% CI = 0.53–0.93; *P* < .00001). Subgroup analysis further showed RDW ≥ 14.0 on admission could be regarded as a predictive morbidity factor for ARDS (SMD = 1.36; 95% CI = 0.66–2.07; *P* = .0002), and RDW ≥ 15.5 on admission could be also regarded as a predictive mortality factor for ARDS (SMD = 0.73; 95% CI = 0.49–0.97; *P* < .00001).

**Conclusion:**

RDW levels seems to be a useful tool for predicting the morbidity and mortality of critically ill patients with ARDS.

## 1. Introduction

Acute respiratory distress syndrome (ARDS) refers to acute diffuse lung injury caused by various intrapulmonary and extrapulmonary pathogenic factors and the development of acute respiratory failure. Despite recent advances in limited fluid resuscitation, prone ventilation, and protective lung ventilation,^[1,2]^ the mortality rate of ARDS is up to 40%.^[3]^ The early diagnosis of ARDS can be treated as soon as possible and significantly reduce mortality. Acute physiology and chronic health evaluation II (APACHE-II) score, simplified acute physiology score and sequential organ failure assessment score, which are used to assess the prediction of critically ill patients, but has poor specificity for ARDS. Therefore, it is important to identify inflammatory markers or comprehensive indicators that predict ARDS.^[4]^

Red blood cell distribution width (RDW) is an indicator that reflects the volume dispersion of red blood cells and measures the size variability of red blood cells, and increases in pulmonary and systemic inflammation, which is involved in the development and prognosis of ARDS.^[5,6]^ Recent studies^[7]^ have shown a significant relationship between RDW and the risk of morbidity and mortality in patients with multiple diseases. Some studies have revealed that RDW is related to the prediction of critically ill patients, including patients with ARDS and sepsis.^[8–10]^ Therefore, this meta-analysis is the first to explore the predictive value of RDW for ARDS. This result may help formulate clinical guidelines to guide clinicians to detect ARDS as soon as possible and make judgments on its prognosis.

## 2. Methods

### 2.1. Search strategy

Three researchers independently searched 5 online electronic databases (PubMed, Embase, Medline, Weifang, and CNKI) from January 1, 2000, to August 1, 2024 with the following keywords: “acute respiratory distress syndrome”, “ARDS”, “acute lung injury” or “ALI” and “Red blood cell distribution width” or “RDW”.

### 2.2. Inclusion and exclusion criteria

Randomized controlled trials, cross‐sectional studies, prospective and retrospective comparative cohort studies, controlled clinical trials, case series with a control group and case‐control studies, assessing RDW upon admission in ARDS patients were carefully searched for inclusion. The primary outcome was prediction of morbidity and mortality. Studies lacking full - text availability or a proper control group were excluded.^[11,12]^

### 2.3. Study selection

After removing duplicate articles, three researchers independently screened all available abstracts to identify and exclude irrelevant articles. Then, the full text of the eligible articles was screened according to the inclusion and exclusion criteria. The predefined criteria for data review/assessment can reduce the likelihood of bias and obviate the need to include all confounding variables in the context and outcome measures, study endpoints, and/or dataset interpretations. This approach ensures that the selection process is systematic and transparent, minimizing the potential for arbitrary decisions that could introduce bias.^[13]^ If a consensus could not be reached, the disagreement was escalated to a fourth researcher with extensive experience in meta-analysis and critical care medicine. This fourth researcher acted as an arbitrator and made the final decision based on a thorough review of the relevant literature and the specific criteria outlined in our study protocol. Specifically, the fourth researcher reviewed the full text of the disputed articles, assessed their relevance to our research question, and ensured that the decision was consistent with our predefined inclusion and exclusion criteria. This rigorous process helped us maintain the integrity and consistency of our study selection and data extraction.

### 2.4. Data extraction

F-FH and L-NY used predetermined keywords to extract the following data from the included studies: first author, publication year, age, country, type of study, sample size, primary disease and outcome. For continuous data, the mean and standard deviation (SD) are extracted. When the mean and SD are not present, but the median and interquartile range are provided, the mean is estimated by Luo^[14]^ method and SD is estimated by the Wan^[15]^ method. Ethical approval was not required, as this meta-analysis involves secondary data analysis from published studies without direct patient involvement.

### 2.5. Data synthesis

We conducted this meta-analysis using Review Manager version 5.4. The mean differences for continuous data along with the 95% confidence interval (CI) were evaluated according to the Cochrane Handbook for Systematic Reviews of Interventions.^[16]^ The *I*^2^ statistics are used to assess statistical heterogeneity, a value >50% was considered to have significant heterogeneity. The sensitivity analysis was repeated and the impact of each study in the study was evaluated by deleting different studies each time. Publication bias was assessed using the Begg test and Egger regression test. The Egger regression test is particularly sensitive to small-study effects and provides a quantitative assessment of asymmetry, while the Begg test is a nonparametric approach that examines the rank correlation of the standardized effect sizes.

### 2.6. Quality of included studies

Three researchers independently used the Newcastle Ottawa Scale^[17]^ to assess the quality of each included study. It focuses on 3 areas: comparability, choice and outcome. If the total score is 8 or 9 stars, it is considered to be high quality research; If the total score is 6 or 7 stars, it is considered to be a moderate quality study; If the total score is <5 stars, it is considered a low-quality study. Any disagreement should be resolved through negotiation with fourth researcher with extensive experience in meta-analysis and critical care medicine.

## 3. Results

### 3.1. Basic characteristics

**Figure 1.**
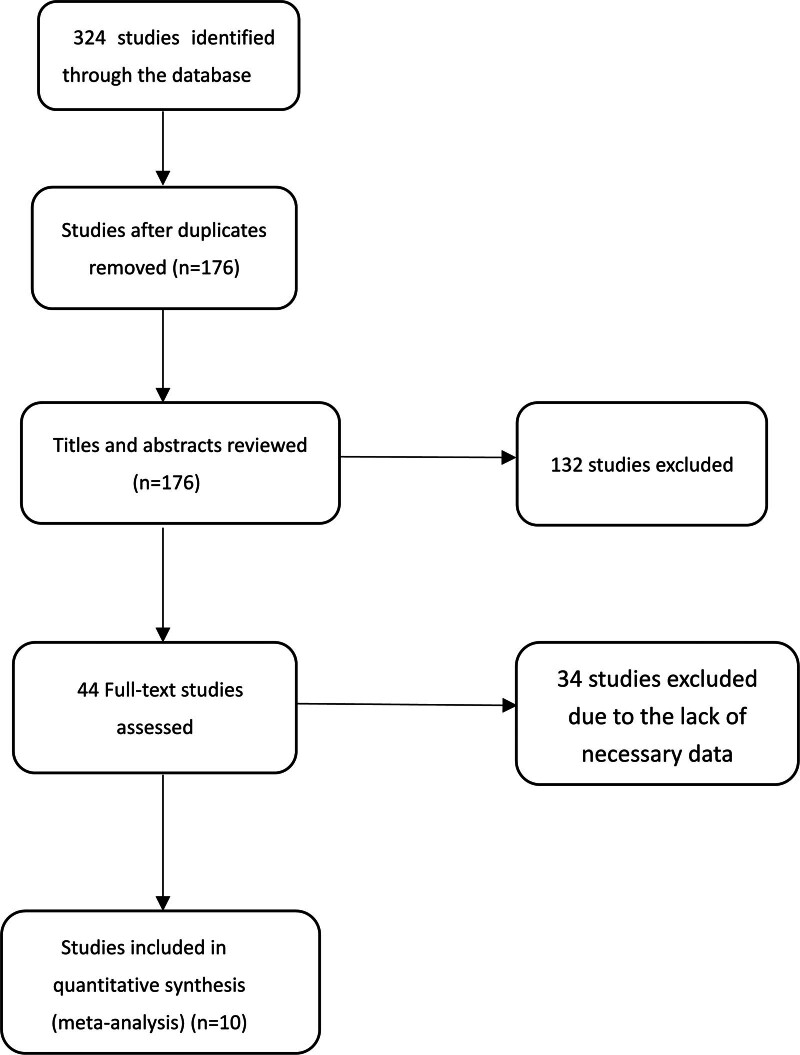
The selection process for the studies that were incorporated into the research.

Ten studies^[18–27]^ out of 324 identified articles were incorporated based on the inclusion and exclusion criteria (Fig. 1, Table 1). Four^[18–21]^ articles assessed RDW levels on admission to evaluate between ARDS groups and non-ARDS groups, others^[22–27]^ discussed RDW levels on admission between non‐survivors and survivors.

**Table 1:** Characteristics of studies.

Study	Publication year	Age (yr)*	Male (%)*	Country	Type of study	Study size	Primary disease	Outcome
Peng et al^[18]^	2015	43.3 ± 14.9	22 (68.8%)	China	Retrospective	152	Acute pancreatitis	Patients with high RDW remained significant difference compared with non-APALI patients (*P* = .003)
Ye et al^[19]^	2018	–	–	China	Retrospective	120	Eighteen cases of severe pneumonia, 14 cases of severe multiple injuries, 11 cases of severe pancreatitis, 7 cases of acute peritonitis, 5 cases of acute suppurative cholangitis, 3 cases of hemorrhagic shock, 3 cases of pesticide poisoning, 2 cases of drowning, and 1 case of purulent meningitis	RDW in ARDS group was higher than that in control group (*P *< .05)
Xiao et al^[20]^	2019	45 ± 9.5	105 (73.4%)	China	Retrospective	610	Severe burns	RDW were significantly higher in ARDS patients than non-ARDS patients (*P* < .01)
Han and Guo^[21]^	2021	56.6 ± 21.0	6 (50.0%)	China	Retrospective	60	Acute pancreatitis	RDW in ARDS group was significantly higher than that in non-ARDS group (*P *< .05)
Lv et al^[22]^	2020	68 ± 22.0	57 (70.0%)	China	Retrospective	86	Eight cases of acute severe pancreatitis, 63 cases of lung infection, 6 cases after operation, 3 cases of poisoning, 4 cases of cerebral hemorrhage, 1 case of trauma, and 1 case of acute myocardial infarction.	The RDW in the death group was significantly higher than that in the survival group within 24 hours after admission (*P *< .05)
Yoo et al^[23]^	2020	74 ± 2.9	112 (70.9%)	South Korea	Retrospective	228	NA	The RDW difference between the survival group and the non-survival group was not statistically significant (*P* = .67)
Zhou and You^[24]^	2020	80.32 ± 8.5	28 (50.0%)	China	Retrospective	58	Sepsis	There was significant difference in RDW between survival group and death group (*P* < .05)
Tang et al^[25]^	2021	67.52 ± 15.0	99 (56.6%)	China	Retrospective	842	Trauma	The deceased group had higher RDW than the survivor group. (*P* = .001)
Wang et al^[26]^	2021	69 ± 10.0	30 (60.0%)	China	Retrospective	96	NA	The RDW in the death group was significantly higher than that in the survival group (*P* < .001).
Alkhatib et al^[27]^	2020	67.0 ± 16.8	65 (57.5%)	USA	Retrospective	318	NA	AUC for the base model without RDW was 0.76, and 0.78 following the addition of RDW (*P* = .048)

APALI = acute pancreatitis–associated lung injury, ARDS = acute respiratory distress syndrome, AUC = area under curve, NA = not available, RDW = red blood cell distribution width .

* ARDS group.

### 3.2. Main results

#### 3.2.1. Prediction of morbidity

**Figure 2.**
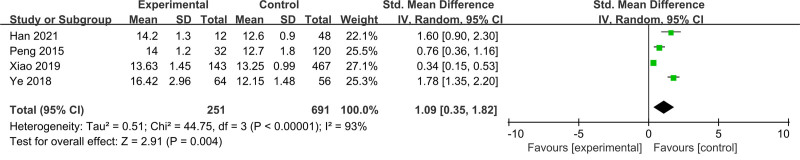
Forest plot for the influence of red blood cell distribution width on the morbidity associated with acute respiratory distress syndrome. This forest plot compares experimental (ARDS) and control (non-ARDS) groups across 4 studies. Each study’s data is presented with mean values, SD, and total sample sizes for both groups. The SMD with 95% CI is calculated using the IV random-effects model, accounting for heterogeneity (*I*^2^ = 93%). The pooled effect size, indicated by the diamond, shows a significant overall effect (Z = 2.91, *P* = .004) favoring the experimental group. ARDS = acute respiratory distress syndrome, CI = confidence interval, IV = inverse variance, SD = standard deviation, SMD = standard mean difference.

**Figure 3.**
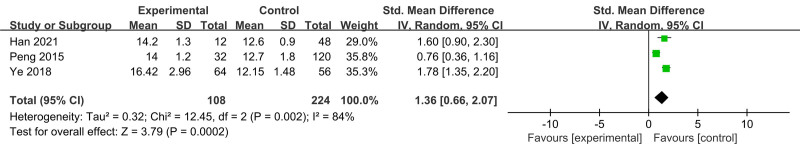
Subgroup analysis of the impact of red blood cell distribution width levels at or above 14.0 on morbidity in acute respiratory distress syndrome. This forest plot compares experimental (ARDS) and control (non-ARDS) groups across 3 studies. Each study’s data is presented with mean values, SD, and total sample sizes for both groups. The SMD with 95% CI is calculated using the IV random-effects model, accounting for heterogeneity (*I*^2^ = 84%). The pooled effect size, indicated by the diamond, shows a significant overall effect (Z = 3.79, *P* = .0002) favoring the experimental group. ARDS = acute respiratory distress syndrome, CI = confidence interval, IV = inverse variance, SD = standard deviation, SMD = standard mean difference.

Four articles with a total of 942 participants were evaluated for the morbidity of ARDS. Elevated RDW levels on admission was significantly associated with the morbidity risk of ARDS (standardized Mean Difference [SMD] = 1.09; 95% CI = 0.35–1.82; *P* = .004) (Fig. 2). Subgroup analysis of 3 studies showed that RDW ≥ 14.0 on admission could be regarded as a predictive morbidity risk for ARDS. (SMD = 1.36; 95% CI = 0.66–2.07; *P* = .0002) (Fig. 3).

#### 3.2.2. Prediction of mortality

**Figure 4.**
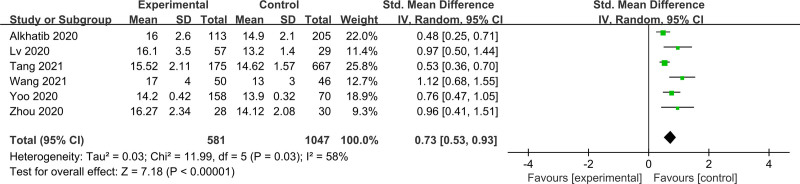
The effect of red blood cell distribution width on mortality in patients with acute respiratory distress syndrome. This forest plot compares experimental (non-survivors) and control (survivors) groups across 6 studies. Each study’s data is presented with mean values, SD, and total sample sizes for both groups. The SMD with 95% CI is calculated using the IV random-effects model, accounting for heterogeneity (*I*^2^ = 58%). The pooled effect size, indicated by the diamond, shows a significant overall effect (Z = 7.18, *P* < .000 01) favoring the experimental group. CI = confidence interval, IV = inverse variance, SD = standard deviation, SMD = standard mean difference.

**Figure 5.**
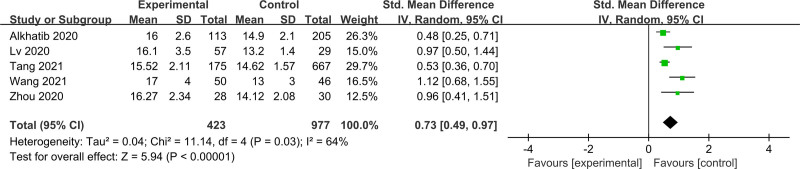
Subgroup analysis of the effect of red blood cell distribution width levels at or above 16.0 on mortality in acute respiratory distress syndrome. This forest plot compares experimental (non-survivors) and control (survivors) groups across 6 studies. Each study’s data is presented with mean values, SD, and total sample sizes for both groups. The SMD with 95% CI is calculated using the IV random-effects model, accounting for heterogeneity (*I*^2^ = 64%). The pooled effect size, indicated by the diamond, shows a significant overall effect (Z = 5.94, *P* < .00001) favoring the experimental group. CI = confidence interval, IV = inverse variance, SD = standard deviation, SMD = standard mean difference.

Six studies with a total of 1310 participants were assessed for the mortality of ARDS. Elevated RDW levels on admission was also significantly associated with the mortality of ARDS (SMD = 0.73; 95% CI = 0.53–0.93; *P* < .00001) (Fig. 4). Subgroup analysis of 5 studies showed that RDW ≥ 15.5 on admission could be regarded as a predictive mortality factor for ARDS (SMD = 0.73; 95% CI = 0.49–0.97; *P* < .00001) (Fig. 5).

### 3.3. Sensitivity analysis

Although significant heterogeneity was found in studies predicting morbidity and mortality, the sensitivity analysis showed that the results were robust, and its significance was not affected by omitting each included study from the pooled analysis. The Begg test (*P*>.05) and Egger regression test (*P*>.05) were all used to analyze the risk of publication bias in the study, indicating that our pooled analysis did not have significant publication bias.

### 3.4. Quality assessment

Table 2 summarizes the results of the quality assessment. Methodological quality was rated as high in 4 studies and moderate in 6 studies.

**Table 2:** Methodological assessment according to the Newcastle–Ottawa scale.

Study	Selection				Comparability	Outcome			Total score†
	Representativeness	Selection	Ascertainment	Outcome of interest		Assessment	FU	Adequacy of FU	
Peng et al^[18]^	*	*	*	*	**	*	–	–	7
Ye et al^[19]^	*	*	*	*	*	*	–	–	6
Xiao et al^[20]^	*	*	*	*	**	*	*	–	8
Han and Guo^[21]^	*	–	*	*	**	*	–	–	6
Lv et al^[22]^	–	*	*	*	**	*	*	–	7
Yoo et al^[23]^	*	*	*	*	**	*	*	*	9
Zhou and You^[24]^	*	–	*	*	*	*	*	–	6
Tang et al^[25]^	*	*	*	*	**	*	*	*	9
Wang et al^[26]^	*	*	–	*	*	*	*	–	6
Alkhatib et al^[27]^	*	*	*	*	*	*	*	*	8

– indicates no stars.

FU = follow-up.

† We considered a study to be of high quality when the total score was 8 or 9 stars, moderate quality when the total score was 6 or 7 stars, and low quality when the total score was 5 stars or fewer.

## 4. Discussion

In our study, we found that elevated RDW levels can predict not only the morbidity of ARDS, but also predict the mortality of ARDS. In subgroup analysis, RDW ≥ 14.0 can be used as a cutoff for predicting the morbidity of ARDS, and RDW ≥ 15.5 can be used as a cutoff for predicting the mortality of ARDS. Therefore, the measure of RDW levels may be a simple markers to predict the morbidity and mortality of ARDS.

The potential biological mechanisms underlying this association are multifaceted and primarily involve inflammation and oxidative stress. Inflammation plays a crucial role in the pathogenesis of ARDS. The damage to alveolar epithelial cells and endothelial cells, along with the accumulation of inflammatory cells and increased production of pro-inflammatory cytokines, are key factors in ARDS development. Inflammatory cytokines can inhibit the maturation of red blood cells in the bone marrow, leading to the release of a large number of immature reticulocytes into the bloodstream. This process increases RDW levels, as these reticulocytes are larger than mature red blood cells. Elevated RDW levels can thus serve as an indirect marker of systemic inflammation, which is closely related to the severity and progression of ARDS.^[28–30]^ Oxidative stress is another important factor contributing to ARDS. Recent research^[31]^ have shown that oxidative stress can lead to poor lung function and increased vascular permeability, which are characteristic features of ARDS. Elevated RDW levels have been associated with increased oxidative stress, likely due to the damage caused to red blood cells by reactive oxygen species.^[32]^ This oxidative damage can affect red blood cell morphology and size, further contributing to the elevation of RDW levels. The interplay between inflammation and oxidative stress creates a vicious cycle that exacerbates lung injury and worsens ARDS outcomes.

Wang et al^[33]^ confirmed elevated RDW levels was associated with the mortality of ARDS in critically ill patients and can be used as an independent predictor of mortality after adjustment for a few clinical characteristics. The ARDS mortality model established by Yu et al^[34]^ showed that higher RDW levels was related to higher 30-day mortality. Ku et al^[35]^ revealed that elevated RDW levels was an independent risk factor for death in patients with gram-negative bacteremia. In subgroup analyses, we found that initial RDW ≥ 14.0 on admission could be regarded as a predictive morbidity risk factor for ARDS and initial RDW ≥ 15.5 was the cutoff for predicting ARDS mortality. There is little research on initial RDW predicting the morbidity risk of ARDS. However, some recent studies have revealed that the initial RDW predicts the mortality of ARDS. After adjustment for potential confounders related to 30-day mortality, the RDW levels ≥ 14.5 were an independent predictor of 30-day mortality (odds ratio [OR] = 1.91; 95% CI = 1.08–3.39; *P* = .02) compared with low RDW levels (<14.5).^[33]^ Similarly, this trend appears in the 90-day mortality (OR = 2.56; 95% CI = 1.50–4.37; *P* = .0006).^[33]^ Yu et al^[34]^ also suggested RDW>15.05 associated with higher 30-day mortality (OR = 2.33; 95% CI = 1.15–4.75, *P* = .019). These studies indicated that RDW levels is closely related to the development and outcome of ARDS. Although there is no consensus on the optimal cutoff value of RDW, it cannot be ignored that the high RDW levels is associated with increased morbidity and mortality in critically ill patients with ARDS.

In our meta-analysis, significant heterogeneity was observed in studies predicting both the morbidity and mortality of ARDS. This heterogeneity may be attributed to several factors. First, differences in patient demographics, such as age, comorbidities, and underlying diseases, could contribute to the variability in RDW levels and their association with ARDS outcomes. Second, study settings, including geographic location and hospital type, may also play a role. Finally, the specific methodologies used in each study could further impact the results. Although sensitivity analysis demonstrated the robustness of our findings, the sources of this heterogeneity were not fully explored. Future research should aim to address these factors more comprehensively to better understand the heterogeneity observed in our analysis. Additionally, the significant heterogeneity we observed may also be attributed to the variability in RDW thresholds across different studies. This variability highlights the challenges in establishing a universal RDW cutoff value for predicting ARDS morbidity and mortality. Different cutoff values may lead to varying sensitivities and specificities in different patient populations, which could potentially affect the clinical utility of RDW as a predictive marker. Therefore, future research should focus on identifying more consistent RDW cutoff values through large-scale prospective studies. This approach could enhance the clinical applicability of RDW as a prognostic tool for ARDS.

### 4.1. Strengths and limitation

This is the first attempt to use meta-analysis to prove the predictive value of RDW for the morbidity and mortality of ARDS, which may help to provide strong evidence for arguments established in the literature. Although our studies showed significant heterogeneity, the included studies were assessed as moderate or high quality. Luo^[14]^ and Wan^[15]^ methods are widely accepted and provided rational estimates when converting data, they may introduce some degree of bias due to assumptions made during the conversion process. This could potentially affect the precision of our pooled estimates, and we acknowledge this as a limitation of our study. The included studies were all retrospective, which may affect the accuracy of the results. Therefore, larger prospective studies should be conducted to validate our results. More importantly, we roughly estimated the cutoff value of RDW for ARDS prediction, more large-scale studies will be encouraged in the future to find the cutoff value of RDW.

## 5. Conclusion

RDW levels seems to be a useful tool for predicting the morbidity and mortality of critically ill patients with ARDS. Further well-designed researches, especially large-scale prospective researches, are needed to prove the predictive value of RDW.

## Author contributions

**Conceptualization:** Qiong Wang, Li-Na Yan, Xin Chen.

**Data curation:** Li-Na Yan, Xin Chen.

**Formal analysis:** Yong Song, Wei-Na Du, Bei-Bei Wang, Qiong Wang, Li-Na Yan, Xin Chen.

**Funding acquisition:** Li-Na Yan, Xin Chen.

**Investigation:** Yong Song, Wei-Na Du, Bei-Bei Wang, Qiong Wu, Li-Na Yan, Xin Chen.

**Methodology:** Fei-Fei Hou, Bei-Bei Wang, Qiong Wang, Qiong Wu.

**Project administration:** Fei-Fei Hou, Yong Song, Wei-Na Du, Bei-Bei Wang, Qiong Wu, Li-Na Yan, Xin Chen.

**Resources:** Bei-Bei Wang, Qiong Wang, Qiong Wu.

**Software:** Fei-Fei Hou, Bei-Bei Wang, Qiong Wang, Qiong Wu, Xin Chen.

**Supervision:** Wei-Na Du, Qiong Wang, Qiong Wu.

**Validation:** Fei-Fei Hou, Yong Song, Bei-Bei Wang, Qiong Wang, Qiong Wu.

**Visualization:** Fei-Fei Hou, Bei-Bei Wang, Qiong Wu, Xin Chen.

**Writing – original draft:** Fei-Fei Hou, Yong Song, Wei-Na Du, Qiong Wang, Xin Chen.

**Writing – review & editing:** Fei-Fei Hou.
